# The St. John's Hospital Case: A Legal Synopsis

**Published:** 1913-06-28

**Authors:** 


### THE ST. JOHN'S HOSPITAL CASE.

### A Legal Synopsis.

BY A LAWYER.

This case has aroused such general interest in the hospital world that there is a distinct danger of the scope of the judgment delivered by Mr. Justice Pickford and its effect being distorted out of all recognition by lay opinion.

According to the reports of the case, which appeared in the *Times* newspaper of the 18th, 19th, and 20th inst., the plaintiffs, who are members of the medical staff of the hospital, claimed two things—viz.:

(1) Damages for wrongful dismissal from their position as members of the medical staff, and

(2) An injunction to restrain the hospital from excluding them from its membership.

It appears that the hospital consists of a body of members incorporated under the Companies Acts, with articles of association and rules drawn up for the guidance of those responsible for the administration of its affairs. The plaintiffs were in fact members of the hospital in its corporate legal capacity, and the second question for decision was, broadly speaking, whether in view of the evidence of tension existing between them and the senior physician the hospital was justified in excluding them from its membership. The learned Judge appears to have held that there was nothing in the rules which would justify the hospital in taking this course. He conceded, however, that in the event of misconduct having been pleaded or proved by the hospital there might be good ground for their membership being forfeited; but the question of misconduct was never even raised in the couse of the case.

Point number one is of far greater interest to the medical profession and the institutional world than the somewhat academic question whether members of a corporation can or cannot be excluded from the privileges which they may enjoy in their capacity as such. How far is a general hospital in the absence of any definite written contract or binding rules entitled to dismiss the medical staff with or without notice? Expert evidence was taken on this point, and great weight must necessarily attach to Sir Thomas Barlow's pronouncement on the subject. His opinion, based on many years' experience, is that in the absence of rules limiting the tenure of the medical staff, and apart from serious misconduct found after proper inquiry, the tenure of the medical staff is permanent and not determinable by notice. This appears to be the generally accepted view of all cases where no definite written contract of service has been entered into between the individual members of the staff and the institution which they serve. It appears to have been adopted by Mr. Justice Pickford, who added incompetence as a further ground for dismissal. The learned Judge held that there could not be such a right, as was claimed by the St. John's Hospital, to terminate the appointments of the medical staff at any moment, thereby implying that even if misconduct or incompetence had been alleged or proved, reasonable notice of some kind would have been necessary.

It remains to be seen how far the Court of Appeal will go towards supporting this decision.

